# Evidence of autoimmune etiology in children with small fiber neuropathy

**DOI:** 10.3389/fneur.2026.1927782

**Published:** 2026-10-01

**Authors:** Morgan Rook, Fredrick Perry, Aalif Zaheer, Shaista Safder, Vikram Prakash

**Affiliations:** 1 Florida State University College of Medicine, Tallahassee, FL, United States; 2 Department of Pediatrics, Cardinal Glennon Children’s Hospital/St. Louis University, St. Louis, MO, United States; 3 Center for Digestive Health and Nutrition, Arnold Palmer Hospital for Children, Orlando, FL, United States; 4 Leon Pediatric Neuroscience Center of Excellence, Arnold Palmer Hospital for Children, Orlando, FL, United States

**Keywords:** autoimmune, dysautonomia, gastrointestinal dysmotility, IVIG, neuropathic pain, pediatric neurology, small fiber neuropathy

## Abstract

**Objective:**

Small fiber neuropathy (SFN) is becoming increasingly recognized as a cause of autonomic dysfunction, sensory disturbances, and neuropathic pain. While SFN was previously considered predominantly idiopathic, cases of autoimmune SFN are now well described in adults but remain underexplored in children.

**Methods:**

In this retrospective chart review, we identified pediatric patients diagnosed with SFN of suspected autoimmune etiology. SFN was diagnosed based on epidermal nerve fiber density (ENFD) and/or sweat gland nerve fiber density (SGNFD) values from skin biopsy and/or quantitative sudomotor axon reflex testing (QSART). Autoantibodies were assessed using the Mayo Clinic Dysautonomia Panel and the Washington University in St. Louis (WUSTL) Sensory Neuropathy Panel.

**Results:**

Twenty patients with SFN of suspected autoimmune etiology were identified. Seventy-five percent were females, with mean ages at symptom onset and diagnosis of 11.158 and 14.038 years, respectively. Presenting symptoms included gastrointestinal (GI) symptoms (60%, *n* = 12), neurological/sensory (40%, *n* = 8), and postural orthostatic tachycardic syndrome (POTS) (40%, *n* = 8); 35% (*n* = 7) presented with a combination of symptoms. IgM TS-HDS antibodies were identified in 45% (*n* = 9), IgG FGFR3 antibodies in 25% (*n* = 5), both antibodies in 5% (*n* = 1), and ganglionic acetylcholine receptor antibodies in 5% (*n* = 1). Comorbid autoimmune conditions included Crohn’s disease (*n* = 1), and Sjögren’s syndrome (*n* = 3). Abnormal findings were recorded on QSART in 5 of 6 patients and GI motility studies in 10 of 11 patients. Twelve patients received IVIG therapy for more than 3 months, of whom 10 (83%) reported improvement in symptom burden or complete resolution of symptoms.

**Conclusion:**

Pediatric SFN typically presents with a combination of POTS, neurologic, and GI symptoms. Our study demonstrates a potential autoimmune etiology, supported by the presence of identifiable autoantibodies and observed clinical improvement after a minimum of 3 months of IVIG therapy.

## 1 Introduction

Small fiber neuropathy (SFN) is a peripheral neuropathy that predominantly affects the small Aδ-fibers and unmyelinated C-fibers involved in autonomic and sensory functions of the nervous system (1), causing neuropathic pain, altered sensation, changes in sweating, orthostatic hypotension, and bowel and bladder dysfunction (2–4). While many conditions have been found to be associated with SFN, including diabetes mellitus, nutritional deficiencies, inherited sodium channel disorders, hemochromatosis, and toxic exposures such as chemotherapy or chronic alcohol use, the majority of cases of SFN are otherwise idiopathic or cryptogenic (5, 6).

Autoimmunity has been increasingly investigated as an etiology of cryptogenic SFN (4, 7). Autoantibodies have been found to be associated with SFN (8, 9), especially in those presenting with symptoms of dysautonomia (10). In one study of 155 patients with SFN, IgM autoantibodies to trisulfated heparan disaccharide (TS-HDS) and IgG antibodies to fibroblast growth factor receptor 3 (FGFR3) were found in 37 and 15% of adult patients, respectively (11). Similarly, in a later study of 322 adults with SFN, 28% had elevated IgM TS-HDS, and 17% had elevated IgG FGFR3 (12). In adults, the presence of ganglionic acetylcholine receptors (gAchR) antibodies, first associated with autoimmune autonomic neuropathies in 2000 (13), was found in up to 50% of patients with autoimmune autonomic ganglionopathy (14) and likely plays a role in autonomic dysfunction in SFN (15). Additionally, higher rates of autoimmune diseases, including Sjögren’s disease and sarcoidosis, among others, have been reported in adult patients with SFN compared with the general population (5, 7, 16, 17).

While there is no consensus on the clinical criteria for diagnosis of SFN, several methodologies are available for assessing patients with suspected disease. ENFD on skin biopsy of the distal leg is commonly considered a gold-standard test for the diagnosis of SFN and can be measured and compared with published normative data. Destruction of peripheral nerve fibers results in a lower total ENFD for those with SFN. Autonomic tests, such as thermoregulatory sweat testing are frequently used in diagnosis of SFN. Quantitative sudomotor axon reflex test (QSART) is a more accessible test that measures sweat production in response to cholinergic agents and does not require the same degree of environmental control (18).

Treatment of autoimmune SFN consists of symptomatic management and immunomodulatory treatments. Intravenous immunoglobulin (IVIG), a commonly used treatment, is proposed to improve symptoms of autoimmune SFN through several immune-modulating mechanisms (19). The literature on IVIG for the treatment of SFN has shown mixed results. While symptomatic improvement has been reported in some cases and several open-label studies (20–22), recent randomized clinical trials have failed to find meaningful improvements in symptoms (23) or significant changes in nerve biopsy results (24).

While SFN has been more widely explored in the adult population it has been underreported and understudied in the pediatric population. We previously described the GI manifestations of pediatric SFN (25) while others have noted possible autoimmune associations (26). In this study, we aim to further characterize the clinical presentations of SFN in the pediatric population and provide further evidence supporting a potential autoimmune etiology.

## 2 Methods

With the approval of the Institutional Review Board at Orlando Health Arnold Palmer Hospital for Children, we conducted a retrospective chart review of patients identified from the Child Neurology Clinic at the Orlando Health Children’s Neuroscience Institute and the Orlando Health Center for Digestive Health and Nutrition at Arnold Palmer Hospital for Children who were diagnosed with SFN of a suspected autoimmune etiology. To be included in this study patients had to be aged 0–21 years at the time of the SFN diagnosis and have confirmed autoimmune autoantibodies or a comorbid autoimmune condition at the time of diagnosis. As this study was retrospective, all participants also required a waiver of consent/assent from the Institutional Review Board unless they were still active patients at the clinic at the time of the chart review, in which case appropriate written informed consent and assent were obtained. Upon consent/assent, all available data from the patient’s initial workup, detailed below, and subsequent follow-up, including demographics, clinical characteristics, and diagnostic testing, were collected for analysis.

As described in our previous study (25), all patients and/or their parents completed a 12-point review-of-systems questionnaire asking about symptoms in multiple organ systems as part of the original workup for SFN. All patients in whom SFN was considered underwent the same initial screening laboratory evaluation, including a complete blood count (CBC), comprehensive metabolic panel (CMP), thyroid-stimulating hormone (TSH), thyroxin (T4), complement components 3 and 4 (C3/C4), copper, ceruloplasmin, zinc, ferritin, selenium, vitamin B12, folate (vitamin B9), vitamin D, vitamin B1, vitamin B6, homocysteine, methylmalonic acid, erythrocyte sedimentation rate (ESR), C-reactive protein (CRP), carnitine, acylcarnitine, thyroid peroxidase antibodies (TPO), thyroglobulin antibodies, anticardiolipin antibodies, and antinuclear antibodies (ANA).

Autoimmune antibody testing, which included the Dysautonomia Panel (Mayo Clinic Laboratories) and the Sensory Neuropathy Panel (Washington University in St. Louis), was requested and obtained depending on insurance coverage and/or authorization. Genetic testing, which included the Comprehensive Neuropathies panel (Invitae) and/or whole-exome sequencing, was also obtained depending on insurance coverage and/or authorization.

Children who were suspected to have neuropathy based on clinical presentation underwent 3-mm punch skin biopsies performed at the distal leg (calf) and lateral thigh using standard techniques at the Orlando Health Child Neurology Clinic at the Leon Pediatric Neuroscience Center of Excellence (27, 28). Specimens were processed at Therapath™ Neuropathology. A 3-mm punch biopsy 10 cm proximal to the lateral malleolus is the most commonly used method. Immunohistochemistry and bright-field microscopy is used to quantify the intraepidermal nerve fiber (IENF) density of the sample. This value is then compared against age- and sex-matched controls (27, 29). The lack of published ENFD values in pediatric patients poses challenges in interpreting biopsy results (30, 31). However, it has been reported that ENFD values in children tend to be higher than in adults, as they decrease with increasing body surface area and age (32, 33).

Due to the lack of published normal ENFD values for this population, we collected and reported age-matched controls for some patients who underwent skin biopsies for investigation of neuropathy but did not meet diagnostic criteria for ENFD. We compared the ENFD from the left distal leg (calf), as this was the site most consistently reported by the Therapath™ laboratory. ENFD values below the fifth percentile adjusted for age and sex are available for comparison for patients older than 18 years of age and are provided by the processing laboratory (27, 33).

GI motility evaluation included a gastric emptying scan, a timed abdominal transit study using SITZMARKS® (Konsyl Pharmaceuticals, Inc.), and manometric evaluations, including antroduodenal, esophageal, colonic, and anorectal manometry.

Patients who complained of orthostatic symptoms were evaluated either by Pediatric Cardiology at an outside institution by tilt-table and cardiovagal testing or by the Orlando Health Child Neurology Clinic at the Leon Pediatric Neuroscience Center of Excellence using orthostatic vital signs. The criteria for diagnosis of postural orthostatic tachycardia syndrome (POTS) were an increase in heart rate of more than 30 beats/min (or more than 40 beats/min in children aged 12–19 years) or a heart rate greater than 120 beats/min after a change of position from supine to standing (head-up tilt) within 10 min of the change in position. Blood pressure and symptoms were also recorded during the testing. Patients who did not undergo tilt-table and cardiovagal testing were diagnosed with POTS if they had a persistent elevation in heart rate that met these criteria and was reproducible on repeated orthostatic vital-sign measurements across multiple patient encounters. Aside from orthostasis, autonomic dysfunction was defined as symptoms arising from parasympathetic or sympathetic dysregulation, including enteric, genitourinary, and thermoregulatory symptoms that caused some degree of patient discomfort.

### 2.1 IVIG

The standard protocol for IVIG was 1 g/kg IVIG daily for 2 days, followed by 1 g/kg of IVIG every 28 days. IVIG treatment continued for a mean of 8.67 months, ranging from 3 to 21 months.

### 2.2 Analysis

The dataset collected in this retrospective chart review was largely descriptive and not well suited to inferential statistical analysis. Instead, data were characterized by percentages and fractions and summarized using descriptive statistics, including means, standard deviations, and ranges. No features of the dataset were assessed for statistical significance.

## 3 Results

### 3.1 Demographics and presentation

Twenty patients with SFN of suspected autoimmune etiology were identified. Fifteen of 20 participants (75%) were female, and the average age at diagnosis was 14.04 years (SD, 3.7; range, 5–17.5 years). The average age at symptom onset was 11 years (SD, 3.8), with an average interval of 2 years between symptom onset and diagnosis; however, one patient experienced symptoms for 9 years before receiving a diagnosis of SFN.

Acute onset of symptoms, defined as full manifestation of the described symptoms within a period of 4 weeks, was seen in 55% (11 of 20) of patients, while the other 45% (9 of 20) had a more gradual progression of symptoms. GI manifestations were a common symptom at presentation, identified in 60% (12 of 20) of patients, while orthostatic dizziness, or POTS was an initial presenting symptom in 40% (8 of 20), and neurologic symptoms were found in another 40% (8 of 20) of patients. Approximately one-third (7 of 20) of patients presented with some combination of two or more symptoms, such as orthostatic dysregulation and GI symptoms or sensory neuropathy and GI symptoms.

#### 3.1.1 GI symptoms

Constipation was the most frequently reported GI symptom (50%, 10 of 20), followed by abdominal pain (30%, 6 of 20), nausea (25%, 5 of 20), early satiety (15%, 3 of 20), and vomiting (10%, 2 of 20). Eleven patients underwent GI motility testing, 10 of whom had one or more abnormal studies, including esophageal manometry, anorectal manometry, gastric emptying, gastric emptying scans, colonic motility, or antroduodenal manometry, for a total of 18 abnormal studies (Table 1).

**Table 1:** Results of GI motility studies.

Gastro-intestinal study	Abnormal motility study	Normal motility study	Total number of studies performed
Esophageal manometry	1	1	2
Anorectal manometry	3		3
Gastric empty scan	6	1	7
Colonic motility	5	2	7
Antroduodenal manometry	3		3
Total number abnormal GI motility studies, *n* (%)	18 (86%)		
Total number of GI motility studies performed, n	21		

Twelve patients underwent multiple GI motility studies, totaling 21 studies. Of the 21 studies, 18 (86%) were abnormal, consistent with some degree of observed GI dysmotility.

#### 3.1.2 Neurologic symptoms

While sensory or neurologic-related symptoms were reported as the presenting symptom in 40% (8 of 20) of patients, several patients continued to have progression of symptoms following initial presentation. Ten patients initially presenting without sensory symptoms developed sensory deficits over the course of their disease. Accounting for the initial eight patients with sensory symptoms and the 10 who subsequently developed them, a total of 18 patients, accounting for 90% (18 of 20) of our study population, experienced some form of sensory alteration. Of those 18 patients, 10 reported numbness and tingling, 14 reported neuropathic pain, six reported both neuropathic pain and numbness and tingling, and four reported non-neuropathic pain.

Length dependent symptoms were present in nine patients, while the other nine patients had non-length-dependent involvement (Tables 2–4). Impaired sensation of the distal leg was found in four subjects. Impaired pinprick sensation was found in four subjects. Midline splitting was found in two participants with impaired pinprick sensation, both of whom showed weakness on the corresponding side.

**Table 2:** Description of sensory symptoms, quality vs. length dependency.

Neurologic symptom	Length dependent pattern	Non-length dependent pattern	Total
Neuropathic pain	8	6	14
Non-neuropathic pain	1	3	4
Numbness	6	4	10
Total	9^b^	9^b^	Total patients with sensory disturbance: 18^a^

a Eighteen total patients with neurologic symptoms. Six patients had both neuropathic pain and numbness, while another four had both non-neuropathic pain and numbness, explaining why the total number of symptom categories adds up to 28.

b Given that multiple patients were counted in multiple categories, the most accurate totals, after subtracting the six patients duplicated in the neuropathic pain group and the four patients duplicated in the non-neuropathic pain group, are nine and nine, respectively.

**Table 3:** Description of sensory symptoms, quality vs. timing.

Neurologic symptom	Persistent	Episodic	# Of patients endorsing symptom
Neuropathic pain	4	10	14
Non-neuropathic pain	2	1	4
Numbness and tingling	3	7	10
			Total number of patients: 18^a^

a Eighteen total patients with neurologic symptoms. Six patients had both neuropathic pain and numbness, while another four had both non-neuropathic pain and numbness, explaining why the total number of symptom categories adds up to 28.

**Table 4:** Description of sensory symptoms, quality vs. distribution.

Neurologic symptom	Diffuse	Limb predominance	Trunk predominance	Total
Neuropathic pain	5	8	1	14
Non-neuropathic pain	1	2	1	4
Numbness	2	6	2	10
Total number of patients				18^a^

Eighteen total patients with sensory disturbance were categorized into three main symptom groups: neuropathic pain (*n* = 14), non-neuropathic pain (*n* = 4), and numbness (*n* = 10).

a Eighteen total patients with neurologic symptoms. Six patients had both limb-predominant neuropathic pain and numbness and were therefore counted in each category of sensory symptom, explaining why the total number of entries adds up to 24.

Atypical presentations in our patient population included one subject with persistent left-sided weakness, and another patient with acute-onset, episodic weakness lasting minutes to hours at a time.

#### 3.1.3 POTS/orthostatic dizziness

Eight patients presented with orthostatic dizziness and were diagnosed with POTS using tilt-table testing (two of eight) or changes in orthostatic vital signs (six of eight) recorded in the clinic. One additional patient went on to develop POTS during the course of the disease.

#### 3.1.4 Psychiatric symptoms

Mood and behavioral problems were present at the time of SFN diagnosis in 11 of the 20 patients. Comorbid diagnoses included four patients with anxiety, three with major depressive disorder, two with mixed anxiety and depression, one with attention-deficit/hyperactive disorder (ADHD), and one with unspecified disordered eating.

#### 3.1.5 Bladder symptoms

Three patients reported impaired sensation of bladder fullness and overflow incontinence.

#### 3.1.6 Headaches

Nine patients reported headache and met the criteria for migraine. Eight patients developed headache after the onset of dysautonomia symptoms. One patient had headaches that did not meet the criteria for migraine prior to the onset of dysautonomia symptoms. None of the patients reported persistent postural-perceptual dizziness (PPPD).

### 3.2 Diagnostic investigations

#### 3.2.1 Autonomic testing

Six patients completed QSART, and five had results suggestive of SFN. As above, tilt-table testing was diagnostic of POTS in both patients who underwent evaluation.

#### 3.2.2 Laboratory testing

Patients underwent the standardized laboratory panel previously described in the Methods. The only significant results were elevated ESR and positive ANA, TPO, and thyroglobulin antibody levels. Abnormal results were found for ANA (1), ESR (3), TPO antibodies (1), C3/C4 (1), folate (1), vitamin D (1), PLP (1), pyridoxic acid (2), and CRP (1).

#### 3.2.3 Skin biopsy

Eighteen of 20 subjects in this study had ENFD values below the reported range for an 18-year-old (11.9 for females and 10.9 for males). Of the subjects with higher values, one had abnormal QSART suggestive of SFN, and the other had an ENFD of 12.68 at the calf, which, according to the literature, was still far below the expected value for the patient’s age and was deemed diagnostic of SFN (27).

#### 3.2.4 Antibodies and comorbid autoimmune diseases

IgM TS-HDS antibody was detected in 10 patients, IgG FGFR3 in 6 patients, and both antibodies in 1 patient. One patient was positive for ganglionic neuronal acetylcholine receptor antibody (AchR N-ganglionic Ab). One subject was transiently positive for a myasthenia gravis–blocking antibody but never developed myasthenic symptoms.

Of note, three patients in this study were found to have Sjögren’s syndrome. One patient was diagnosed with Sjögren’s syndrome prior to the onset of neuropathic symptoms and was later found to have skin biopsy findings and IgM TS-HDS antibody serology suggestive of SFN. Two patients initially presenting with neuropathy were diagnosed with comorbid Sjögren’s syndrome after developing additional findings, such as sicca syndrome and serologic markers. Additionally, one patient had uncontrolled Crohn’s disease at the time of SFN onset.

#### 3.2.5 Comorbid neurologic and musculoskeletal diagnoses

The following significant diagnoses were found to be comorbid with SFN in study participants: Muckle–Well’s syndrome (*n* = 1), epilepsy (*n* = 2), Tourette’s syndrome (*n* = 1), alternating hemiplegia of childhood (*n* = 1), benign hypermobility (*n* = 2), and EDS (*n* = 1).

#### 3.2.6 Genetic testing

Genetic testing was performed on 11 patients; whole-exome sequencing (WES) was performed in 10 patients, and the Invitae laboratory neuropathy panel was performed in one subject. No pathogenic neuropathy-associated genes were identified; however, two patients had variants of unknown significance in RANBP2, a gene known to be associated with autosomal dominant familial acute necrotizing encephalopathy (34). Additionally, one patient was found to have pathogenic variants in RYR1, a gene known to be associated with congenital myopathies and malignant hyperthermia (35). Another had variants in CACNA1A and CACNA1S genes, which are known to encode voltage-gated calcium channels and have been association with hemiplegic migraines and muscular pathologies, including hypokalemic periodic paralysis and congenital myopathies (36, 37).

## 4 IVIG treatment and outcomes

At the time of data collection, 15 patients had initiated IVIG treatment using the standard protocol of 1 g/kg IVIG daily for 2 days, followed by 1 g/kg IVIG every 28 days. Twelve patients had completed 3 or more months of IVIG, and three had completed less than 3 months of treatment. Ten of the 12 patients had clinical improvement in their symptoms (Table 5). Duration of therapy ranged from 3 to 21 months, with a mean duration of 8.67 months and a median of 6 months. Throughout the course of treatment, total IVIG doses ranged from 4 to 22 g/kg, with a median of 7 g/kg.

**Table 5:** Symptom improvement after minimum 3 months IVIG therapy.

Symptom	# Of patients with symptom receiving IVIG treatment, *n*	# Of patients with symptom improvement after IVIG, *n* (%)
Pain/numbness/tingling	12	10 (83%)
GI symptoms	10	8 (80%)
POTS	6	5 (83%)
Bladder symptoms	2	1 (50%)
Total	12	10 (83%)

A total of 12 patients received at least 3 or more months of IVIG. Of the 12 patients represented here, n is the denominator or the number of patients with a specific symptom and *n* (%) is the number of patients with symptom improvement. Multiple patients presented with a combination of symptoms so they were counted in multiple symptom categories however each patient was counted only once in the final total.

Response to IVIG was generally measured clinically based on patient history, self-reported symptoms, and physical examination findings at follow-up visits. Pain symptoms were assessed by subjective changes in the frequency or severity of pain, impact on activities of daily living, and need for analgesia. GI symptoms were assessed by patient-reported frequency or severity of symptom burden and the need for medications such as prokinetic and anti-nausea medications or parenteral nutrition. POTS symptoms were assessed based on patient-reported changes in the frequency of presyncopal symptoms, impact on activities of daily living, and need for medications.

Of the 12 patients who completed at least 3 months of IVIG treatment, seven of eight patients reporting headache, three of three subjects with length-dependent sensory symptoms, and seven of nine with non-length-dependent symptoms reported improvement, described as reduced frequency, reduced severity, or complete resolution of numbness, tingling, or pain. For example, one patient initially presenting with persistent sensory symptoms despite treatment of comorbid Sjögren’s disease received IVIG treatment for SFN and had complete resolution of her symptoms, including weakness and left-sided hemianesthesia.

Ten of the 12 patients treated with IVIG presented with GI manifestations. Eight of these 10 patients experienced a reduction in symptoms during IVIG treatment. Notably, one patient with severe dysautonomia and whole-colon dysmotility had a total return of colonic motility and was able to transition from total parenteral nutrition (TPN) to meeting 50% of her nutritional needs through a GJ tube.

Additionally, six of the 12 patients treated with IVIG had symptoms of POTS. Five of these six patients experienced improvement in POTS symptoms, such as fewer presyncopal episodes, after a minimum of 3 months of IVIG.

Objective laboratory data, such as ENFD, were repeated in only one participant and showed recovery of nerve fiber density from 7.31 to 14.43 in the left thigh and from 5.14 to 12.5 in the calf, with ENFD values changing from those consistent with SFN to values consistent with non-SFN after 6 months of IVIG treatment.

### 4.1 Side effects of IVIG

Of the 12 patients with suspected autoimmune SFN that underwent 3 or more months of IVIG therapy, 10 experienced adverse effects. The most severe adverse effect reported was mast cell activation syndrome following the initial IVIG infusion, which presented as severe hypotension and respiratory failure. Notably, following a desensitization protocol, IVIG was otherwise well tolerated for the remainder of treatment. At various points during the course of treatment, nine of 12 patients experienced aseptic meningitis, reported as mild (one of nine), moderate (six of nine), or severe (two of nine). Mild reactions did not require any intervention. Moderate reactions were managed with hydration, a three-day course of steroids, famotidine, diphenhydramine, and ibuprofen/acetaminophen. Following a moderate or severe reaction, subsequent doses were premedicated with a similar protocol to that used for moderate postinfusion reactions. Severe reactions resulted in hospital admission for symptom management and division of subsequent IVIG treatment into two sessions.

### 4.2 Follow-up

Duration of follow-up ranged from 3 to 47 months, with a median follow-up duration of 22 months.

## 5 Discussion

Small fiber neuropathy has been increasingly recognized as a cause of pain, autonomic dysfunction, and sensory disturbances in children. As more data have become available, many patients previously misdiagnosed with functional or idiopathic pain syndromes, including fibromyalgia or amplified musculoskeletal pain syndrome (AMPS), are now receiving the diagnosis of SFN (30, 31, 38). The lack of universal, standardized diagnostic criteria and a lack of clinician awareness of SFN have contributed to misdiagnosis, stigmatization, and diagnostic delay. In our patient population, the average time to diagnosis was 2 years, with one patient experiencing symptoms for as long as 9 years before receiving a diagnosis. This diagnostic delay often contributed to significant symptom progression and reduced quality of life, which might otherwise have been minimized with prompt diagnosis. With further characterization of the presentation of autoimmune SFN, more accessible testing modalities, and improved clinician awareness, we may be able to reduce diagnostic delays and ultimately reduce morbidity associated with SFN.

Recognizing the complex and variable constellation of signs and symptoms is the key to making the diagnosis of SFN. At disease onset, the most commonly reported classes of symptoms were dysautonomic GI dysfunction, seen in 60% (12 of 20) of patients, neurologic symptoms, seen in 40% (8 of 20); and orthostatic dizziness, seen in 40% (8 of 20). Notably, a substantial portion of our patients (35%, 7 of 20) presented with some combination of GI, POTS, or neurologic symptoms. From onset, symptoms often evolved over time. Although not always present initially, the most common symptom throughout the disease course was some form of sensory problem, which was seen in 90% (18 of 20) of our patients.

Many of the symptoms we describe are easily overlooked in favor of alternative diagnoses. For example, two patients who presented with midline-split hemisensory impairment were initially diagnosed with functional neurologic disorder (FND). However, after abnormal skin biopsy findings and confirmation of associated autoantibodies, the patients were diagnosed with SFN. GI symptoms such as constipation, nausea, vomiting, and abdominal pain were common among our patients but were initially attributed in some cases to other comorbid conditions, such as EDS, benign hypermobility, or POTS, and thus went unrecognized as manifestations of SFN. In many of these cases, a neurogenic pattern of dysfunction on motility testing was ultimately helpful in identifying underlying SFN, but such testing is not always readily available. Similarly, incomplete bladder emptying and incontinence are known to occur secondary to constipation and can easily be overlooked as manifestation of SFN. Orthostatic dizziness and POTS were presenting symptoms in our population and are overall common among females in general, especially adolescents (39). As our population had an average age of diagnosis of 14 years and was predominantly female, dysautonomic symptoms in several patients were initially attributed solely to POTS, without consideration of SFN. Finally, while pain and sensory symptoms are frequently reported in patients with SFN, they are often non-specific and do not consistently follow an easily identifiable pattern of distribution. We observed this in our population, with an equal proportion of length-dependent (9 of 18) and non-length-dependent (9 of 18) pain and sensory symptoms. This is consistent with previously described findings for SFN (30, 31).

In developing an illness script for the diagnosis of a disease, knowledge of disease onset and progression is essential to differentiating discrete pathologies. In our population, disease onset and progression were highly variable. Approximately half (55%) of our population had an acute or subacute onset, while the other half (45%) had a more insidious progression of symptoms. An acute or subacute onset has been postulated as an indicator of an autoimmune etiology for SFN (11, 40). However, we failed to observe a predominance of this presentation in our population of children with biopsy-proven SFN.

The etiology of SFN remains idiopathic in a large proportion of children. Autoimmunity has become increasingly recognized as a common cause (30, 31, 41). This study provides further support for a potential autoimmune etiology. Known autoantibodies IgM TS-HDS and IgG FGFR3 were detected in 50% (10 of 20) and 30% (6 of 20) of our selected patients, respectively. There were additionally several comorbid autoimmune diagnoses among our patients: three with Sjögren’s syndrome and one with Crohn’s disease. The observed favorable response among the 12 patients treated with IVIG, including a single participant’s repeat skin biopsy showing normalized ENFD values, may further support an underlying autoimmune etiology and suggest IVIG as a potential therapy. As is well known, small-diameter nerve fibers, unlike large-diameter fibers, have the capacity to regenerate (41); prompt diagnosis and treatment may reduce overall morbidity.

We do, however, acknowledge that the generalizability of this study is limited by selection bias, as participants were selected specifically for the presence of known autoimmune autoantibodies or comorbid autoimmune conditions. Without a control group, the clinical significance of the associated autoimmune autoantibodies remains unclear, as does the association between the antibodies and the observed response to IVIG treatment. Furthermore, the retrospective nature of the study, lack of objective data documenting severity and response to IVIG, and relatively small sample size (*n* = 20) further limits our ability to draw conclusions from this study. Thus, although the observed response appears favorable, the role of IVIG in this population of patients with pediatric SFN remains largely unclear, and the data should be interpreted accordingly, especially because previous IVIG therapy trials have shown mixed results (20–24). Additional randomized controlled trials with objective outcome measures are needed to evaluate the efficacy of IVIG as a treatment for suspected autoimmune SFN.

SFN remains diagnostically challenging in the pediatric population due to the lack of clear diagnostic criteria, including published normal ENFD values for patients younger than 18 years. In this study, a diagnosis of SFN was made if ENFD values from left distal leg (calf) were below the fifth percentile when compared with age- and sex-adjusted values reported by Therapath™ for patients older than 18 years of age (27, 33). In patients who clinically demonstrated features of SFN but had ENFD values above the fifth percentile, QSART was used as an additional diagnostic measure that could further support an objective SFN diagnosis. However, in one patient in this study, both ENFD and QSART did not meet the initial diagnostic criteria until further literature review revealed that the patient’s ENFD value remained abnormal for his age (27). Considering this abnormal ENFD values in the context of the patient’s clinical features, a diagnosis of SFN was made, and the patient was included in the study. This highlights the ambiguity surrounding SFN diagnosis given the lack of standardized diagnostic criteria and age-adjusted ENFD values and the current need for clinical judgment when diagnosing children with SFN.

Given the ongoing challenges surrounding the diagnosis and treatment of pediatric SFN, there is a need for increased clinician awareness of SFN, refined clinical diagnostic criteria, and standardized age-adjusted ENFD values for children to facilitate timely diagnoses, evaluation of validated therapies, and improved outcomes for these patients.

## Data Availability

The datasets presented in this article are not readily available because of the difficulty with presenting the large volume of data. Requests to access the datasets should be directed to Vikram.prakash@orlandohealth.com.
